# Use behavior and spatial characteristics of National Fitness Routes in China: an in situ observational study

**DOI:** 10.3389/fpubh.2026.1904288

**Published:** 2026-09-03

**Authors:** Yunxi Tian, Baoquan Zhu, Mengwei Zhao, Zhengyang Yao, Qisheng Han, Kai Wang

**Affiliations:** 1 School of Horticulture and Landscape Architecture, Henan Institute of Science and Technology, Xinxiang, Henan, China; 2 Logistics Management Office, Henan Institute of Technology, Xinxiang, Henan, China; 3 College of Landscape Architecture and Arts, Northwest A&F University, Yangling, Shaanxi, China

**Keywords:** behavior mapping, *in situ* observation, NFRs, OGs, physical activity

## Abstract

**Background:**

The installation of outdoor gyms (OGs), also known as the National Fitness Route (NFR) program in China, is increasing in popularity as a government-funded solution to declining population physical activity (PA) levels. However, evidence regarding how these facilities are used and whether they effectively support PA remains limited.

**Methods:**

Systematic *in situ* observations were conducted across four representative NFRs in Yangling District, Shaanxi Province, China, during three seasons. Demographic characteristics, behavioral patterns, and OGs use were recorded using the System for Observing Play and Recreation in Communities (SOPARC). Behavior mapping and kernel density estimation were used to examine the spatial distribution of activities, and binary logistic regression was performed to identify factors associated with moderate-to-vigorous physical activity (MVPA).

**Results:**

A total of 9,704 person-observation records were collected, OGs accounted for 15.2% of all observation records, with seniors representing the largest user group. The air walker, leg and waist/back massager were the most frequently used types of OGs. Sitting, standing, and walking were the predominant activities, and behavioral activities exhibited clear spatial clustering around seating areas, dedicated pathways, and open activity spaces. After adjustment for potential confounders, age, time period, week period, temperature, air quality, and site characteristics were independently associated with MVPA, whereas gender and season were not.

**Conclusion:**

OGs use represented only a small proportion of activities observed within NFRs, indicating that these spaces function not only as settings for PA but also as important places for recreation and social interaction. Integrating age-appropriate OGs with walking paths, seating areas, tree shade, and flexible open spaces may better support diverse recreational behaviors and promote PA. These findings provide evidence-based guidance for optimizing the planning and spatial design of NFRs to support healthier and more inclusive public fitness environments.

## Introduction

Although the health benefits of regular physical activity (PA) are well documented, the number of people worldwide who do not meet PA recommendations remains high (1, 2). To address this problem, governments are building new PA spaces for public use. Urban green spaces (UGS) frequently used by the residents for daily activities, are being built with more sports facilities.

Existing research indicates that UGS with sport facilities have effects on levels of PA for different age groups (3–5). A study from a sample with an average age of 37 years old found that soccer and basketball fields in UGS were positively associated with frequency of PA for physically active people (6). Pathways had a positive influence on the duration of moderate-vigorous physical activity (MVPA) for seniors during the park visits (7). More recent evidence from Chengdu, China, further suggests that the built environment is associated with older adults’ walking behavior, with sidewalk provision, population density, and access to medical facilities positively related to walking time. Importantly, these associations varied across spatial locations and levels of walking time, highlighting the spatial and behavioral heterogeneity of built-environment effects on PA (8). Playgrounds in neighborhood parks provided a place for children to engage in MVPA (9).

Outdoor gyms (OGs), also referred to as “outdoor fitness equipment,” “outdoor exercise equipment” and “public fitness facilities” can be used by people of all ages to exercise in UGS including parks and community squares (10–13), installation of OGs in UGS have also been shown to be an effective intervention for increasing PA (14) and benefit to physical health (15, 16). Studies assessing the impact of OGs on PA levels (measured by Metabolic Equivalents, METs) have shown that use of OGs for 1 year resulted in 15% increase in users’ PA levels (17). A controlled six-week experiment showed that using OGs was effective in improving fitness, insulin resistance, and other risk factors of chronic diseases (18). In similar 18 week trial in parks with OGs reported improved balance, strength and physical function for seniors with high risk of falls (19).

The installation of OGs in UGS has gained popularity globally (20). Portugal (21) and Spain (22) have reported rapid increases in OGs in parks. In Chile, the construction of OGs in residential squares is an initiative implemented by local governments ranging from small regional towns to large urban areas (23). Chow’s (24) investigation found that OGs have been installed in more than half of parks in Taipei city. Small cities in Canada (1), Australia (11) and Brazil (25) have also added OGs in parks.

In China, the installation of OGs has become a national policy; the “National Fitness Route (NFR)” program. The program uses sports lottery public welfare funds by sports administrative departments at all levels to build OGs in cities and villages, requiring little space at low cost. Generally, a NFR always contains 5–20 OGs, involving multiple functional types such as muscle-strengthening, flexibility, balance, aerobic machines, and relaxation.

NFR typically provides a venue for the public to exercise due to their easy access and no cost. Since the construction of the first NFR in 1996, it has been aggressively promoted in China. In 2007, the General Administration of Sport invested 30 million Yuan in the construction of 1,000 NFRs across the country; by 2010 the investment had increased to 62.5 million Yuan (26). In recent years, the policies of “National Fitness Plan (2021–2025)” and “Healthy China 2030” have emphasized the construction of National Fitness Public Service System (hereinafter referred to as “System”). NFR, as important material carrier of the System, have been widely installing throughout the country. As of 2024, 110,000 NFRs have been built in China, with capital investment reaching over 50 billion Yuan (27). In 2023, China released an implementation plan for enhancing quality of National Fitness facilities to solve the gap between a growing demand for fitness opportunities and the development of fitness facilities (28). This means that more funds will be invested to the construction of the NFRs in the future, providing an impetus to make the NFR work to its full potential.

Although the NFR has become a publicly known and regularly used public space under significant government funding, there remains a large mismatch between the space design of NFR and the scale and impact of its construction in China. At present, the plan and design process of NFR are dominated by sports departments, and the type and layout of OGs are mostly determined by manufacturers in China (26). The designer also simply identifies specific areas of the NFR when designing the UGS. Consequently, NFR as a widely accepted public activity space, lack professional design guidance (29), and to date, studies on the design of the space where OGs are located are few. More studies have focused on the OGs users’ energy expenditure and activity intensity (30, 31), the impact of OGs on PA levels and physical health (12, 14, 17) and users’ perception of OGs (1, 16).

Nevertheless, as a spontaneous activity, the use of OGs is closely linked to the quality of the space design. The characteristics of behavior in space and time further reflect the attractiveness and quality of the physical environment of the space (32). Therefore, it is important to understand how OGs are being used, by whom, and the role of OGs in the entire NFR, to the design approach to the NFR from a user’s use perspective. This is especially true in this new era where improving the quality of the existing NFRs rather than simply increasing their number has become the focus in China.

Research methods of human behavior in UGS have typically included two approaches: traditional *in situ* observations and volunteered geographic information (32). In the current era of big data analysis, established research methods such as SOPARC (System for Observing Play and Recreation in Communities) has been gradually replaced by mobile phone signaling data that saves time and effort (33). However, mobile phone signaling data are more suitable for large scale venues and may not be representative of the general population, as seniors and children, the main users in UGS, rarely own smartphones. Conventional *in situ* observations are more appropriate for studying the number and behavior of NFR visitors of all ages in UGS.

Behavior mapping, as a method to visualize data, is an effective expression based on the direct observation that simultaneously combines a subject’s spatial location and their behavior by graphical form (34). The physical environment and behavior are connected in time and space, providing a common language for linking design to research (35, 36). This is essential for understanding the impact of design on behavior and provides a direct and effective basis for designing spaces that better meet user needs. Moreover, the use of digital tools such as GIS (geographic information system) in behavior mapping also provides an opportunity for further spatial analysis that can quantitatively explore interactions between environment and behavior. To date, behavior mapping has been mainly applied in settings such as urban parks (32), playgrounds (37, 38), schools (34) and hospital grounds (39). There are few studies on NFRs with OGs using behavior mapping.

The lack of relevant information on NFRs use, coupled with a need to improve space design of existing NFRs while planning new ones using significant government resources, calls for a more detailed analysis of patterns of human use.

To address the gap, this study aimed to characterize the demographic and behavioral patterns of observation records, examine patterns of OGs use and PA intensities, and identify the spatial distribution of behaviors across NFRs using *in situ* observation. Behavior mapping and kernel density estimation were used to examine how different behaviors and activity densities were distributed within each NFR, while multivariable logistic regression was used to assess associations between demographic, temporal, environmental, and site characteristics and MVPA. The findings will provide evidence on age-related patterns of OGs use, the role of OGs within NFRs, and the spatial characteristics associated with different activity patterns, thereby informing the planning and design of NFRs to better promote PA and diverse recreational needs.

## Methods

### Study areas

**Figure 1:**
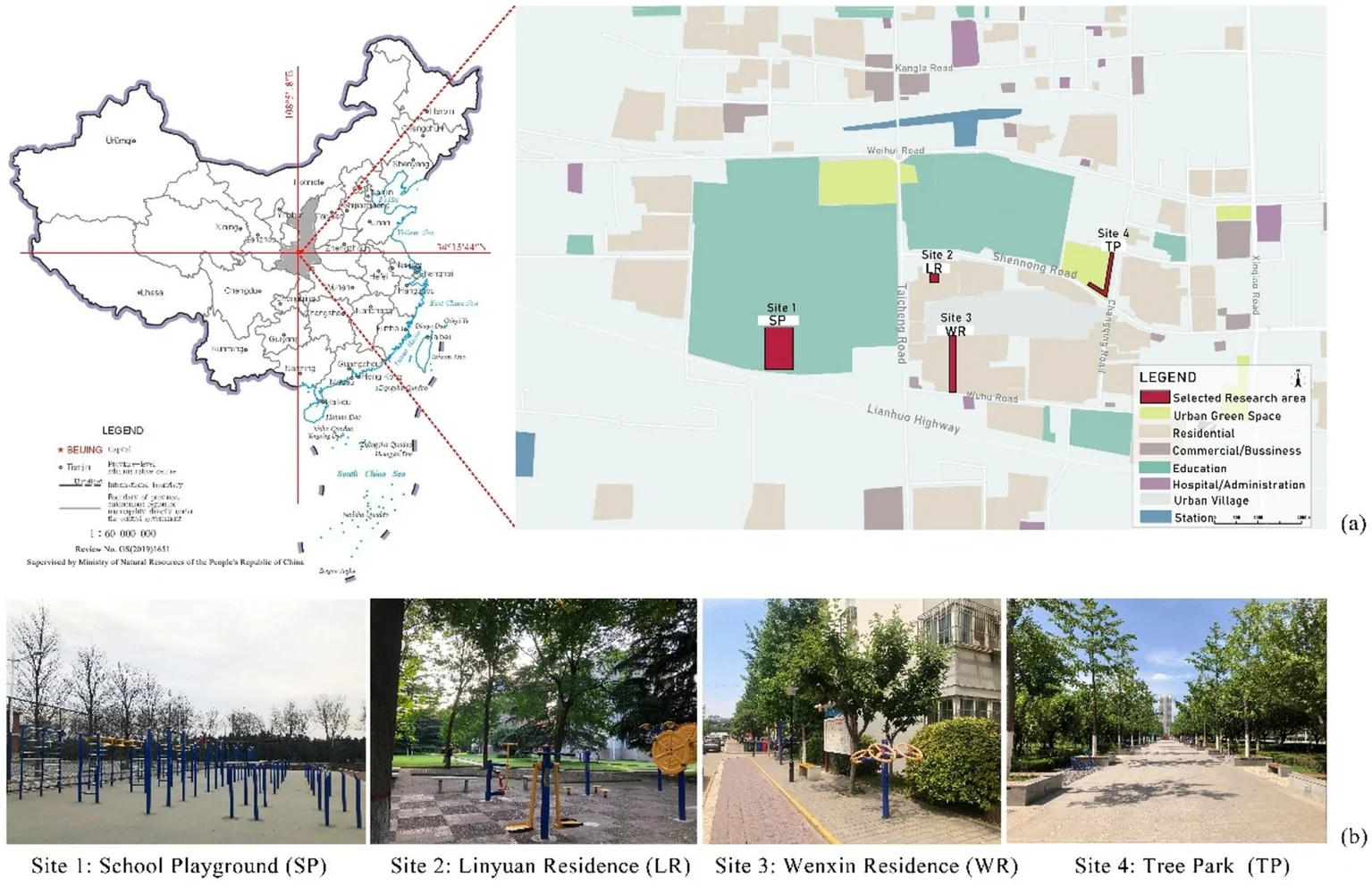
Location of the four sites **(a)** and photographs of the NFRs **(b)**.

In urban areas, NFRs always been installed in various types of UGS including parks, residential green spaces, and attached green spaces. According to China’s Urban Green Space Classification Standard (CJJ/T85-2017) (40), four locations were selected as study areas: Northwest A&F University playground (referred to as school playground, SP), Wenxin residence (WR), Linyuan residence (LR) and tree park (TP). This encompasses three types of UGS with NFR in Yangling agricultural high-tech industry demonstration zone (referred to as Yangling District; Figure 1). Yangling District is located in Shaanxi Province, China, with total built-up area of approximately 16 km^2^ and a total population of 254,600 as of 2023. The *per capita* GDP in Yangling District was 72,768 Yuan in 2023, ranking the fifth in Shaanxi Province (41).

As shown in Table 1, SP has a NFR covering 850 m^2^; its features include sports equipment such as table tennis court and football field, lighting, and no trees. WR has resident population of 4,000 people and an NFR arranged along the main community road that covers 1,260 m^2^. Its features include seats and tables, trees, lighting, and walking paths. LR has resident population of 2,272 people. It has a NFR within the community that is 225 m^2^ with seats and rich vegetation. TP has a 3750 m^2^ NFR arranged along the city roads and connecting the park entrances. Its features include seats, walking paths, and trees. All the NFRs are open all day and allow visitors to enter for free.

**Table 1:** General characteristics of the four NFRs.

NFR	Area/m^2^	Number of OGs	Types of OGs	Type of UGS
SP	850	22	2	attached green space
WR	1,260	20	5	community park
LR	225	12	5	community park
TP	3,750	45	5	comprehensive park

There are five functional types of OGs to meet the exercise needs of the broader population across the four NFRs (Table 2). These include power, flexibility, balance, aerobic machines, and relaxation. All five functional types of OGs were available at WR, LR and TP, whereas SP included only two types: power and flexibility machines.

**Table 2:** Functional types of OGs in the four NFRs.

Functional types	OGs	Graphic icons	SP	WR	LR	TP
Power	Parallel bars	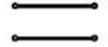	√	√		
	Uneven bars	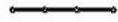	√	√		
	Arm trainer	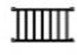	√			
	Seesaw	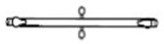				√
	Push-up bar	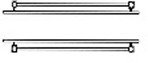				√
	Touching height device	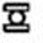	√			
	Single bonny rider	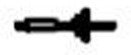		√	√	√
	Pedal power	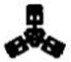			√	
	Double wave plate	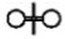			√	√
	Ski machine	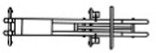				√
	Lower waist trainer	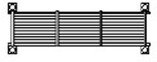		√		
	Waist twister	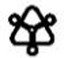		√	√	√
	Sit up bench	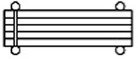		√		√
Flexibility	Wall bars	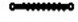	√	√	√	
	Upper body stretch	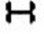		√	√	√
	Double runner	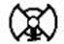		√		
	Stretcher	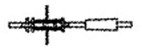				√
Balance	Swing	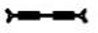		√		
	Tai-chi wheel	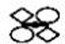		√	√	√
Aerobic	Air walker	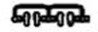		√	√	√
	Seated rowing	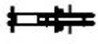			√	√
	Treadmill	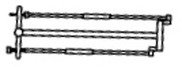				√
Relaxation	Waist/back massager	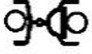		√	√	√
	Leg massager	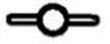			√	√

## Measures

### Observation-SOPARC

**Figure 2:**
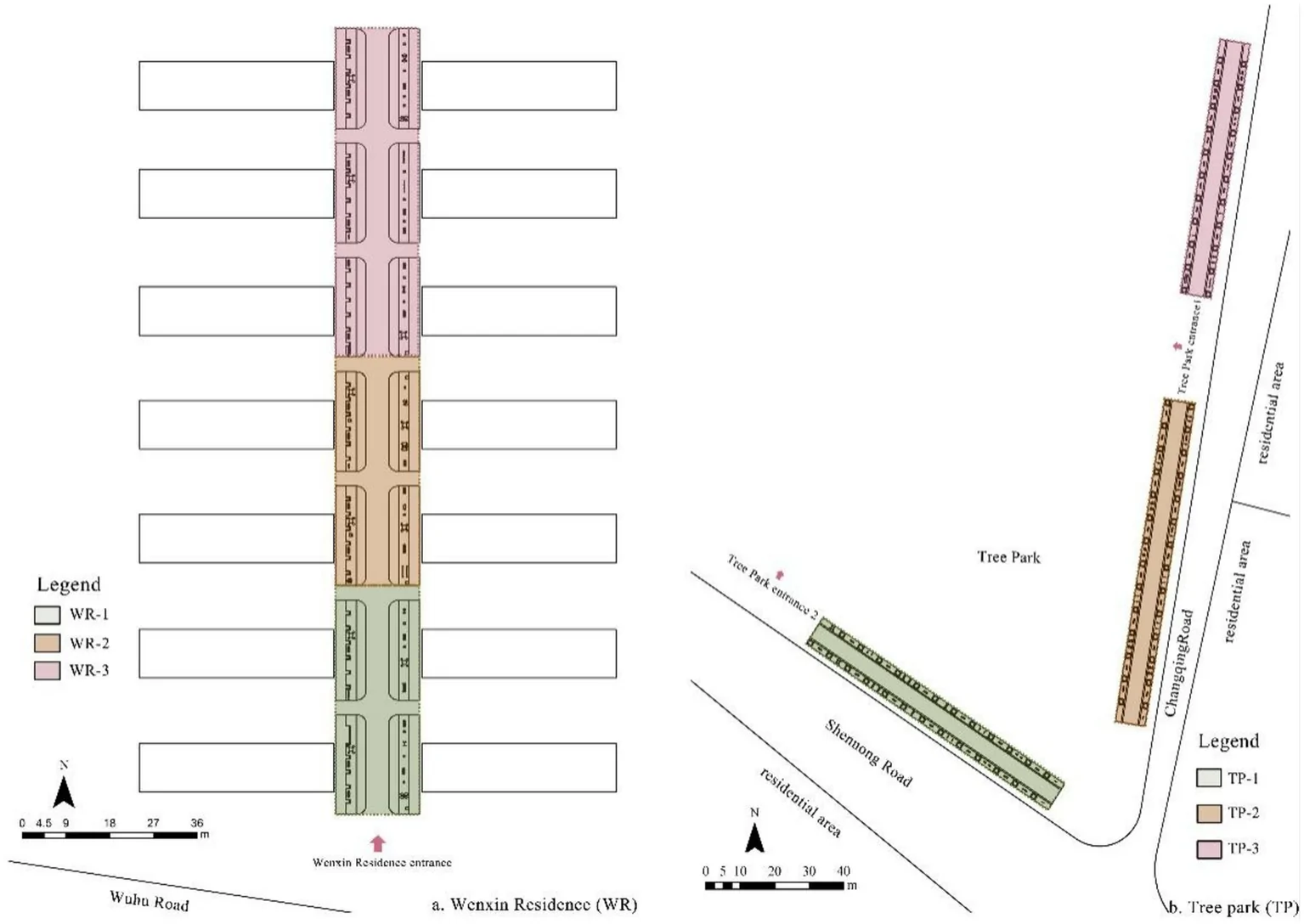
Zoning map of the two NFRs: **(a)** WR; **(b)** TP.

Prior to observations, four NFRs maps were created using GIS. To facilitate accurate observation of records, WR was divided into three sub-areas (WR-1, WR-2, WR-3) and TP was divided into three sub-areas (TP-1, TP-2, TP-3; Figure 2). SP and LR were not zoned due to their small size. Thus, a total of eight observation zones were studied.

The systematic observation of NFRs was based on a modified version of SOPARC, a validated observational tool with reliability exceeding 0.9 (42). For each observation record, gender was recorded and age was estimated and categorized as children (0–12 years), teens (13–20 years), adults (21–59 years), or seniors (≥ 60 years). Daily average Air Quality Index (AQI) scores reported by the Yangling government were recorded during the observation days. An AQI score below 100 indicates that it is suitable for all people to be active outdoors, while scores from 101 to 150 are unhealthy for sensitive people, including children and seniors, in outdoor environments, and scores above 150 are unhealthy for all. The average temperature on the observation day and type of PA were also added to the revised SOPARC but race and social situation of PA were not recorded.

To examine factors associated with PA intensity, the observed behavior categories were further classified into two intensity levels according to their predominant MET values, with reference to the Compendium of Physical Activities and previous studies (43). Behaviors with an estimated intensity of <3 METs were classified as sedentary behavior and light PA (SB/LPA), whereas behaviors with an estimated intensity of ≥3 METs were classified as MVPA. Since the primary objective of this study was to identify factors associated with achieving health-enhancing PA, SB and LPA were combined into a single reference category for binary logistic regression analysis.

Observation data were collected by research assistants during a 24-day observation period in three seasons (summer, autumn, and winter) in 2023. Before collecting data, all assistants completed a training course so that they could learn the definitions of all indicators and familiarize themselves with the observation process and target areas. Training continued until all observers reached a high level of agreement (inter-observer agreement, IOA = 83.2%). Ten assistants were divided into five groups. Each group observed one to two fitness zones. There were four time periods for observation (07: 30–08: 30; 10: 30–11:30; 15:00–16:00; 18:30–19:30) on four weekdays and four weekend days in 2 weeks during each season. Each of the 8 observation zones were observed 96 times (four daily time slots × 8 days × three seasons) in the 24-day observation time.

The unit of analysis in this study was a person-observation recorded during a scheduled SOPARC scan rather than an identified unique visitor. Each scan represented an instantaneous “snapshot” of all individuals present within the observation zone. During each scan, the observer moved through the assigned zone in a fixed direction and recorded each visible person only once, thereby preventing duplicate counts within the same scan. However, because the same individual may have been present during different observation sessions (across time slots, days, or seasons), the same individual could have been recorded in different observation sessions. Individual identities were not collected in accordance with the observational protocol; therefore, repeated observations across separate scans could not be identified or removed. Consequently, the dataset represents repeated person-observations rather than unique individuals, and the results should be interpreted accordingly.

#### Graphical-behavior mapping

During each specific observation period, the observer recorded the behavior in the observation zone without disturbing the visitors as much as possible. The recording method was to reflect the individual behavior in the form of symbols on the corresponding spatial position in the maps. The location and type of OGs as well as the surrounding seating facilities, lighting and trees were also noted. The graphical language combined behavior observations with GIS mapping to create heat and behavior maps directly linked activity density and behaviors to the spatial environment.

### Analysis

We analyzed observation data and spatial patterns. The data analysis process included descriptive statistics, bivariate analysis, and regression analysis. Descriptive statistics were used to summarize the characteristics of observation records, behavioral activities, and observation records involving OGs use across the four NFRs. Differences in demographic, environmental, and temporal variables between the SB/LPA and MVPA groups were examined using chi-square tests. A multivariable binary logistic regression model was fitted using the Enter method, with all prespecified demographic, temporal, environmental, and site variables entered simultaneously. Model fit was evaluated using the omnibus likelihood-ratio test, −2 log likelihood, Cox and Snell *R*^2^, Nagelkerke *R*^2^, and the Hosmer–Lemeshow goodness-of-fit test. Model discrimination was assessed using ROC analysis (AUC), while classification performance was evaluated using overall accuracy, sensitivity, and specificity. Multicollinearity was assessed using variance inflation factors (VIF) and tolerance values. Logistic regression analysis results were presented as the reference categories, regression coefficients, standard errors, an odds ratio (OR) with 95% confidence interval (CI) and the model intercept. All analyses were conducted in SPSS version 26 (IBM Corp, Armonk, NY, United States), and statistical significance was determined at a 0.05 *α* threshold for all analyses.

The graphical analysis was conducted using kernel densities estimation (KDE) in spatial analyst tools in ArcMap 10.4 (ArcGIS © ESRI Redlands, CA). These generated heat maps, which were used to analyze the density of spatial distribution in NFRs. Before the KDE, Average Nearest Neighbor and Ripley’s K function in spatial statistics tools were performed to define the spatial distribution pattern and search radius in KDE. The planar method was applied using an output raster cell size of 0.2 m and a search radius (bandwidth) of 5 m. Observation records were represented as point features, and no population field was specified; therefore, each observation point was assigned equal weight. Kernel density values were expressed as person-observation records per square meter. All analyses were conducted in the WGS 1984 UTM Zone 48 N projected coordinate system to ensure accurate planar distance calculations, and the same KDE parameters were applied across all four study sites to ensure methodological consistency. Because the study sites differed in size and the total number of observation records, the KDE maps were primarily interpreted to identify spatial concentrations within each site rather than to directly compare absolute density values among sites.

## Results

### Descriptive statistics characteristics of observation records in NFRs

A total of 9,704 observation records were collected in the 24 days across three seasons. Among these records, 4,344 (44.8%) were male and 5,360 (55.2%) were female (Table 3). Adults and seniors accounted for the majority of the observed records in the NFRs (82.2%), while the proportion of teens was only 1.2%. Most observations (87.6%) were recorded when the temperature was below 30 °C. During the 24 observation days, the AQI was below 100 on 13 days, accounting for 6,905 (71.2%) observation records. In contrast, the fewest records (*n* = 447) were collected on days when the AQI was classified as unhealthy for all. Among the three seasons, the largest proportion of observation records was collected in summer (59.1%), whereas winter had the fewest. Regarding the daily observation periods, the evening (18:30–19:30) and late-morning (10:30–11:30) sessions each accounted for more than 25% of all records, whereas the afternoon session (15:00–16:00) accounted for fewer than 20%. Observation records on weekdays and weekend days were similarly distributed between weekdays (*n* = 4,886; 50.4%) and weekends (*n* = 4,818; 49.6%).

**Table 3:** Descriptive statistics of observation records across the four NFRs.

Variables		*N* (%)				
		SP (*n* = 459)	WR (*n* = 2,603)	LR (*n* = 291)	TP (*n* = 6,351)	Total (*n* = 9,704)
Gender	Male	263 (57.3)	1,377 (52.9)	134 (46.0)	2,570 (40.5)	4,344 (44.8)
	Female	196 (42.7)	1,226 (47.1)	157 (54.0)	3,781 (59.5)	5,360 (55.2)
Age category	Children	84 (18.3)	516 (19.8)	42 (14.4)	967 (15.2)	1,609 (16.6)
	Teens	8 (1.7)	23 (0.9)	1 (0.3)	82 (1.3)	114 (1.2)
	Adults	338 (73.6)	927 (35.6)	101 (34.7)	3,315 (52.2)	4,681 (48.2)
	Seniors	29 (6.3)	1,137 (43.7)	147 (50.5)	1987 (31.3)	3,300 (34.0)
Temperature	≤10 °C	35 (7.6)	110 (4.2)	26 (8.9)	793 (12.5)	964 (9.9)
	10–30 °C	364 (79.3)	2,179 (83.7)	178 (61.2)	4,822 (75.9)	7,543 (77.7)
	≥30 °C	60 (13.1)	314 (12.1)	87 (29.9)	736 (11.6)	1,197 (12.3)
AQI	≤100 (13 Days)	319 (69.5)	1940 (74.5)	147 (50.5)	4,499 (70.8)	6,905 (71.2)
	101–150 (7 Days)	126 (27.5)	607 (23.3)	132 (45.4)	1,487 (23.4)	2,352 (24.2)
	≥151 (4 Days)	14 (3.1)	56 (2.2)	12 (4.1)	365 (5.7)	447 (4.6)
Season	Summer	238 (51.9)	1,630 (62.6)	182 (62.5)	3,686 (58.0)	5,736 (59.1)
	Autumn	186 (40.5)	863 (33.2)	74 (25.4)	1872 (29.5)	2,995 (30.9)
	Winter	35 (7.6)	110 (4.2)	35 (12.0)	793 (12.5)	973 (10.0)
Time period	07:30–08:30	88 (19.2)	286 (11.0)	83 (28.5)	1906 (30.0)	2,363 (24.4)
	10:30–11:30	35 (7.6)	723 (27.8)	109 (37.5)	1,687 (26.6)	2,554 (26.3)
	15:00–16:00	38 (8.3)	666 (25.6)	38 (13.1)	1,043 (16.4)	1785 (18.4)
	18:30–19:30	298 (64.9)	928 (35.7)	61 (21.0)	1715 (27.0)	3,002 (30.9)
Week period	Weekdays	199 (43.4)	1,365 (52.4)	166 (57.0)	3,156 (49.7)	4,886 (50.4)
	Weekend	260 (56.6)	1,238 (47.6)	125 (43.0)	3,195 (50.3)	4,818 (49.6)

Among the four NFRs, TP accounted for the largest proportion of observation records, representing approximately two-thirds of all records, whereas LR had the fewest (Table 3). Female observation records predominated at LR and TP, while male observation records slightly outnumbered female records at SP and WR. Observation records at the community-based NFRs (WR and LR) were predominantly from seniors, whereas adults constituted the largest age group at SP and TP. Across the four NFRs, similar seasonal and environmental patterns were observed, with comparable distributions across temperature categories, AQI levels, and seasons. Regarding observation periods, more than half of the records at SP were collected during the evening period (18:30–19:30). At TP, more than one-quarter of the observation records were collected during each of three observation periods (07:30–08:30, 10:30–11:30, and 18:30–19:30). At LR, the morning (07:30–08:30) and late-morning (10:30–11:30) observation periods accounted for the largest proportions of records, whereas the morning period represented the smallest proportion of records at WR. Observation records at WR and LR were slightly more common on weekdays, whereas those at SP and TP were more frequently recorded on weekends.

### Behavior characteristics

#### Type of behavior

**Figure 3:**
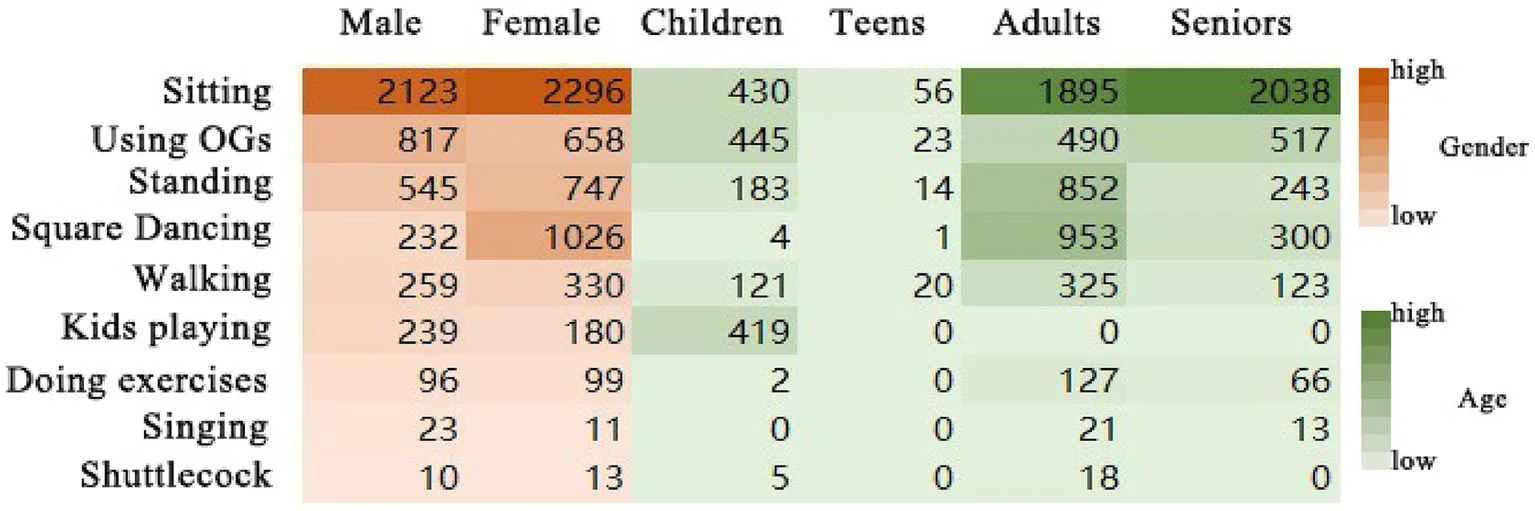
Gender and age distribution among activity behaviors.

During the 24-day observation period from summer to winter, a total of nine types of behaviors were recorded in the four NFRs (Figure 3). Sitting (*n* = 4,419) was the most frequently observed behavior, accounting for 45.5% of all observation records. Using OGs (*n* = 1,475), standing (*n* = 1,292) and square dancing (*n* = 1,258) were the next most common behaviors, representing 15.2, 13.3, and 13.0% of all records, respectively.

Behavioral patterns differed by sex and age group. Female observation records predominated for sitting (*n* = 2,296), square dancing (*n* = 1,026) and standing (*n* = 747), whereas male observation records predominated for sitting (*n* = 2,123) and the use of OGs (*n* = 817). Among the age groups, excluding sitting, OGs use (*n* = 517) was most frequently observed among seniors, whereas standing (*n* = 852) and square dancing (*n* = 953) were most common among adults.

The remaining five behavior categories included walking (*n* = 589), kids playing (*n* = 419), exercising (*n* = 195), singing (*n* = 34), and shuttlecock (*n* = 23). Walking was observed across all age groups and occurred more frequently among females (Figure 3). Kids playing, including pickle ball, skateboarding and chasing each other, was more commonly observed among boys. Doing exercises (including Tai Chi, stretching, fitness exercises), singing (including singing and playing instruments) and shuttlecock (including playing shuttlecock and badminton) were observed almost exclusively among adults.

#### OGs use

Among the 9,704 observation records, 1,475 (15%) involved the use of OGs in the NFRs. Among the five types of OGs, power machines had the largest number of users (*n* = 451), followed by relaxation machines (*n* = 329) and aerobic machines (*n* = 301). Although the power machines included 13 equipment subtypes, the average number of users per subtype was lower than that for relaxation and aerobic machines.

**Figure 4:**
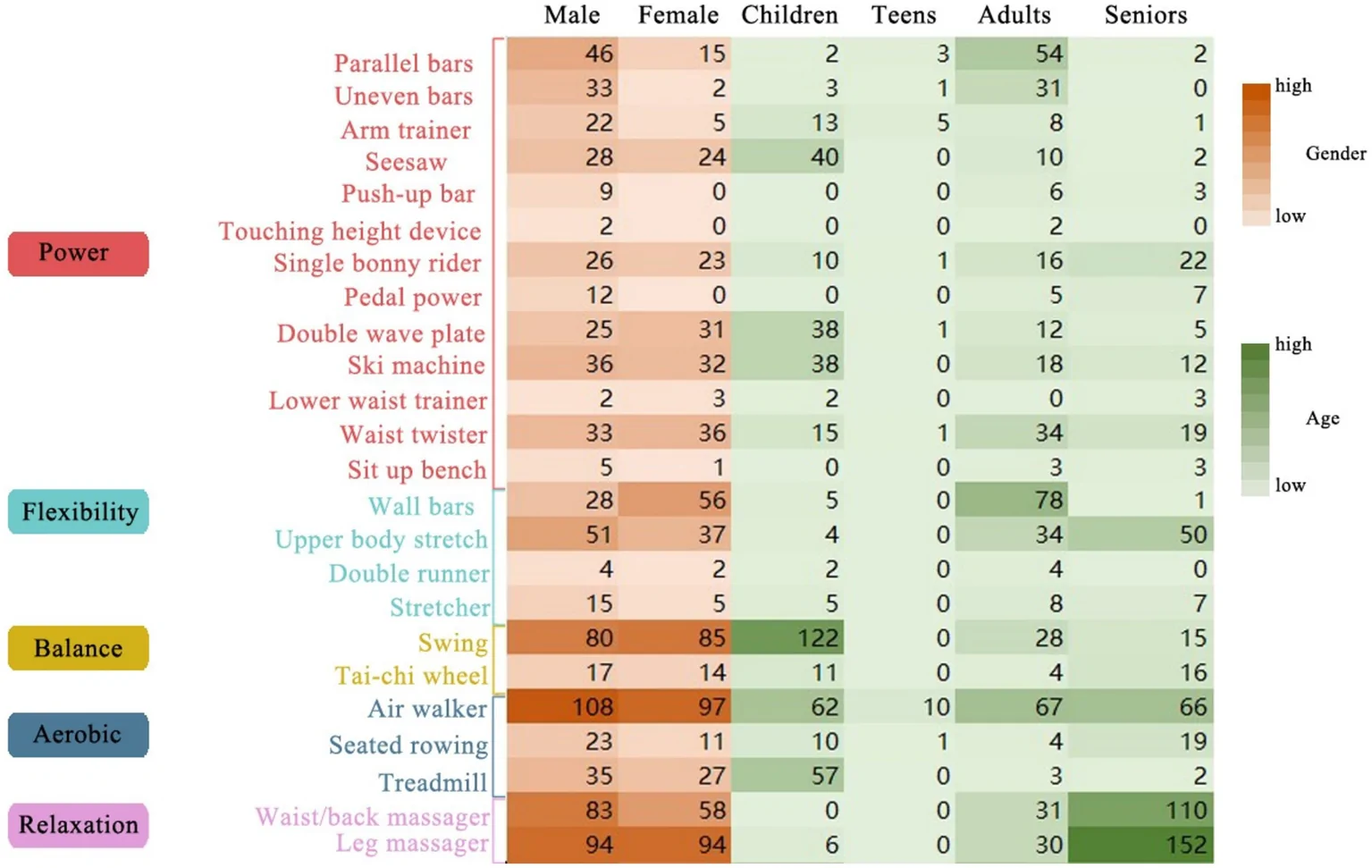
Gender and age distribution among OGs use.

Among individual facilities, the air walker was the most frequently used piece of equipment (*n* = 205), followed by the leg massager (*n* = 188) and the waist/back massager (*n* = 141; Figure 4). In contrast, the touching height device (*n* = 2) was used least frequently.

Male observation records predominated for the use of most OGs, with the exception of the double wave plate and wall bars, which were used more frequently by females. No female observation records were collected using the push-up bar, touching height device and power pedal during the observation period.

Among the age groups, observation records involving seniors accounted for the largest proportion of OGs use, with the leg massagers and waist/ back massagers being the most frequently used equipment. Adults most frequently used the wall bars and parallel bars. The air walker was used across a broader range of age group, including children, adults and seniors. Observation records involving teens accounted for only a small proportion of OGs use, while swings were predominantly used by children.

#### Behavior intensity

Among the 9,704 observation records, 5,798 (59.7%) were classified as LPA/SB, and 3,906 (40.3%) as MVPA. Significant differences between the two PA intensity groups were observed for all study variables (Table 4).

**Table 4:** Key variables of engaging in LPA / SB vs. MVPA.

Variables		*N* (%)		Sample difference
		LPA/SB (*N* = 5,798)	MVPA (*N* = 3,906)	
Gender	Male	2,658 (45.8)	1,686 (43.2)	𝜒^2^ = 6.774 (*p* = 0.009)
	Female	3,140 (54.2)	2,220 (56.8)	
Age category	Children	677 (11.7)	932 (23.9)	𝜒^2^ = 332.618 (*p* < 0.001)
	Teens	58 (1.0)	56 (1.4)	
	Adults	2,783 (48.0)	1898 (48.6)	
	Seniors	2,280 (39.3)	1,020 (26.1)	
Temperature	≤10 °C	453 (7.8)	511 (13.1)	𝜒^2^ = 77.366 (*p* < 0.001)
	10–30 °C	4,648 (80.2)	2,895 (74.1)	
	≥30 °C	697 (12.0)	500 (12.8)	
AQI	≤100 (13 days)	4,160 (71.7)	2,745 (70.3)	𝜒^2^ = 33.08 (*p* < 0.001)
	101–150 (7 days)	1,429 (24.6)	923 (23.6)	
	≥151 (4 days)	209 (3.6)	238 (6.1)	
Season	Summer	3,518 (60.7)	2,218 (56.8)	𝜒^2^ = 74.786 (*p* < 0.001)
	Autumn	1824 (31.5)	1,171 (30.0)	
	Winter	456 (7.9)	517 (13.2)	
Time period	7:30–8:30	1,197 (20.6)	1,166 (29.9)	𝜒^2^ = 227.916 (*p* < 0.001)
	10:30–11:30	1,555 (26.8)	999 (25.6)	
	15:00–16:00	1,312 (22.6)	473 (12.1)	
	18:30–19:30	1734 (29.9)	1,268 (32.5)	
Week period	Weekdays	3,003 (51.8)	1883 (48.2)	𝜒^2^ = 12.004 (*p* = 0.001)
	Weekend	2,795 (48.2)	2023 (51.8)	

Female observation records accounted for a greater proportion of MVPA than male records. Among the age groups, children had a substantially higher proportion of MVPA records than LPA/SB records (23.9% vs. 11.7%), while seniors accounted for a greater proportion of LPA/SB records.

A greater proportion of MVPA records was observed when the temperature was below 10 °C (13.1% vs. 7.8%). Both PA intensity groups were most frequently observed on days with an AQI below 100, However, when the AQI exceeded 151, the proportion of MVPA records was significantly higher than that of LPA/SB records (6.1% vs. 3.6%).

Compared with records classified as LPA/SB, a significantly greater proportion of MVPA records was observed in winter, whereas a significantly smaller proportion was observed in summer. Similarly, a significantly greater proportion of MVPA records was observed during the morning (07:30–08:30) and evening (18:30–19:30), whereas LPA/SB records were proportionally more common during the afternoon period (15:00–16:00). In addition, a slightly higher proportion of MVPA observation records was collected on weekend than on weekdays (51.8% vs. 48.2%).

#### Factors associated with MVPA

A multivariable binary logistic regression model including all prespecified demographic, temporal, environmental, and site variables was fitted using the enter method (Table 5). The overall model was statistically significant (likelihood-ratio *χ*^2^ = 1765.416, df = 17, *p* < 0.001), indicating that the included variables jointly improved the prediction of MVPA compared with the intercept-only model. The model yielded a − 2 log likelihood of 11315.993, a Cox and Snell *R*^2^ of 0.166, and a Nagelkerke *R*^2^ of 0.225. Although the Hosmer–Lemeshow goodness-of-fit test was statistically significant (*χ*^2^ = 322.497, df = 8, *p* < 0.001), the model demonstrated acceptable discrimination, with an area under the receiver operating characteristic curve (AUC) of 0.715 (95% CI: 0.705–0.724, *p* < 0.001). Using a probability threshold of 0.50, the model correctly classified 65.0% of observation records, with a sensitivity of 44.1% and a specificity of 79.2%. Multicollinearity diagnostics indicated no evidence of problematic collinearity among the independent variables (VIF range: 1.018–1.860; tolerance range: 0.538–0.983).

**Table 5:** Binary logistic regression analyses variables predicting MVPA (*N* = 9,704).

Variables		B	SE	Wald	OR (95%CI)
Gender (Ref: female)	Male	−0.035	0.047	0.568	0.965 (0.881–1.058)
Age (Ref: seniors)	Children	1.333	0.072	346.462	3.791 (3.295–4.362) ^***^
	Teens	0.649	0.204	10.156	1.914 (1.284–2.854) ^**^
	Adult	0.145	0.054	7.273	1.156 (1.040–1.284) ^**^
Time period (Ref: 18:30–19:30)	7:30–8:30	0.195	0.062	10.017	1.215 (1.077–1.371) ^**^
	10:30–11:30	−0.227	0.062	13.603	0.797 (0.707–0.899) ^***^
	15:00–16:00	−0.766	0.076	101.662	0.465 (0.401–0.540) ^***^
Week period (Ref: weekend)	weekday	−0.216	0.051	17.823	0.806 (0.729–0.891) ^***^
Temperature (Ref: ≥30 °C)	≤10 °C	0.079	0.347	0.051	1.082 (0.548–2.134)
	10–30 °C	−0.468	0.098	22.953	0.626 (0.517–0.758) ^***^
AQI (Ref: AQI ≥ 151)	≤100	0.403	0.160	6.362	1.496 (1.094–2.046) ^*^
	101–150	−0.004	0.148	0.001	0.996 (0.745–1.332)
Season (Ref: winter)	Summer	−0.487	0.338	2.079	0.615 (0.317–1.191)
	Autumn	−0.351	0.339	1.069	0.704 (0.362–1.369)
NFRs (Ref: TP)	SP	0.530	0.106	24.888	1.700 (1.380–2.093) ^***^
	WR	−1.728	0.066	691.168	0.178 (0.156–0.202) ^***^
	LR	0.242	0.126	3.706	1.274 (0.996–1.630)
Constant		0.355	0.362	0.963	1.426

^***^ Significance at 0.001, ^**^ at 0.01, and ^*^ at 0.05 levels.

After adjustment for all covariates, age, time period, week period, temperature, AQI, and site were significantly associated with MVPA, whereas gender and season were not significantly associated with MVPA (Table 5).

Compared with females, males were not significantly associated with MVPA (OR = 0.965, *p* = 0.451). Compared with seniors, observation records classified as children (OR = 3.791), teens (OR = 1.914), and adults (OR = 1.156) had significantly higher odds of being classified as MVPA.

Using 18:30–19:30 as the reference period, observation records collected during 07:30–08:30 were more likely to be classified as MVPA (OR = 1.215), whereas those collected during 10:30–11:30 (OR = 0.797) and 15:00–16:00 (OR = 0.465) had significantly lower odds of MVPA. Compared with weekend observations, weekday observation records showed lower odds of MVPA (OR = 0.806).

Using ≥30 °C as the reference category, observation records collected at 10–30 °C had significantly lower odds of MVPA (OR = 0.626), whereas no significant difference was observed for temperatures ≤10 °C (*p* = 0.821).

Compared with observation records collected when AQI ≥ 151, those collected when AQI ≤ 100 showed significantly higher odds of MVPA (OR = 1.496), whereas no significant difference was found for AQI 101–150 (*p* = 0.980).

Using TP as the reference site, observation records from SP were more likely to be classified as MVPA (OR = 1.700), whereas those from WR were substantially less likely to be classified as MVPA (OR = 0.178). No statistically significant difference was observed between LR and TP (*p* = 0.054).

Characteristics of NFRs and spatial distribution of observation records.

#### Comparison of OGs use and MET characteristics across the four NFRs

OGs use varied among the four NFRs (Table 6). Although TP recorded the largest number of OGs observation records (*n* = 966), SP exhibited the highest proportion of OGs use, with 43.1% of all observation records involving OGs use. In contrast, WR recorded 261 OGs observation records but had the lowest proportion of OGs use (10.0%). Although LR had the fewest OGs observation records (*n* = 50), it showed the second-highest proportion of OGs use (17.2%).

**Table 6:** OGs use and MET across the four NFRs.

NFRs	OGs use *N* (%)	Total MET	MET per observation record	MET density (MET/m^2^)
SP	198 (43.1)	1506.2	3.5	1.8
WR	261 (10.0)	4,182	1.6	3.3
LR	50 (17.2)	696.6	2.1	2.3
TP	966 (15.2)	17473.9	2.7	4.6
Total	1,475 (15.2)	23858.7	2.5	3.9

Total MET represents the sum of the estimated MET values for all observation records at each NFR; MET per observation record was calculated as the total MET divided by the total number of observation records; MET density was calculated as the total MET divided by the area (m^2^) of each NFR.

Although the total MET expenditure at SP was lower than that at TP and WR, SP had the highest MET per observation record (3.5) among the four NFRs. Among the two community-based NFRs, WR had a substantially higher total MET expenditure (4182) than LR (696.6); however, the MET per observation record was lower at WR (1.6) than that at LR (2.1). The MET per observation record at both community-based NFRs was also lower than the overall study average. TP exhibited the highest MET density (4.6 MET/m^2^), followed by WR, whereas SP had the lowest MET density among the four NFRs.

#### Distribution of OGs observation records across the four NFRs

**Figure 5:**
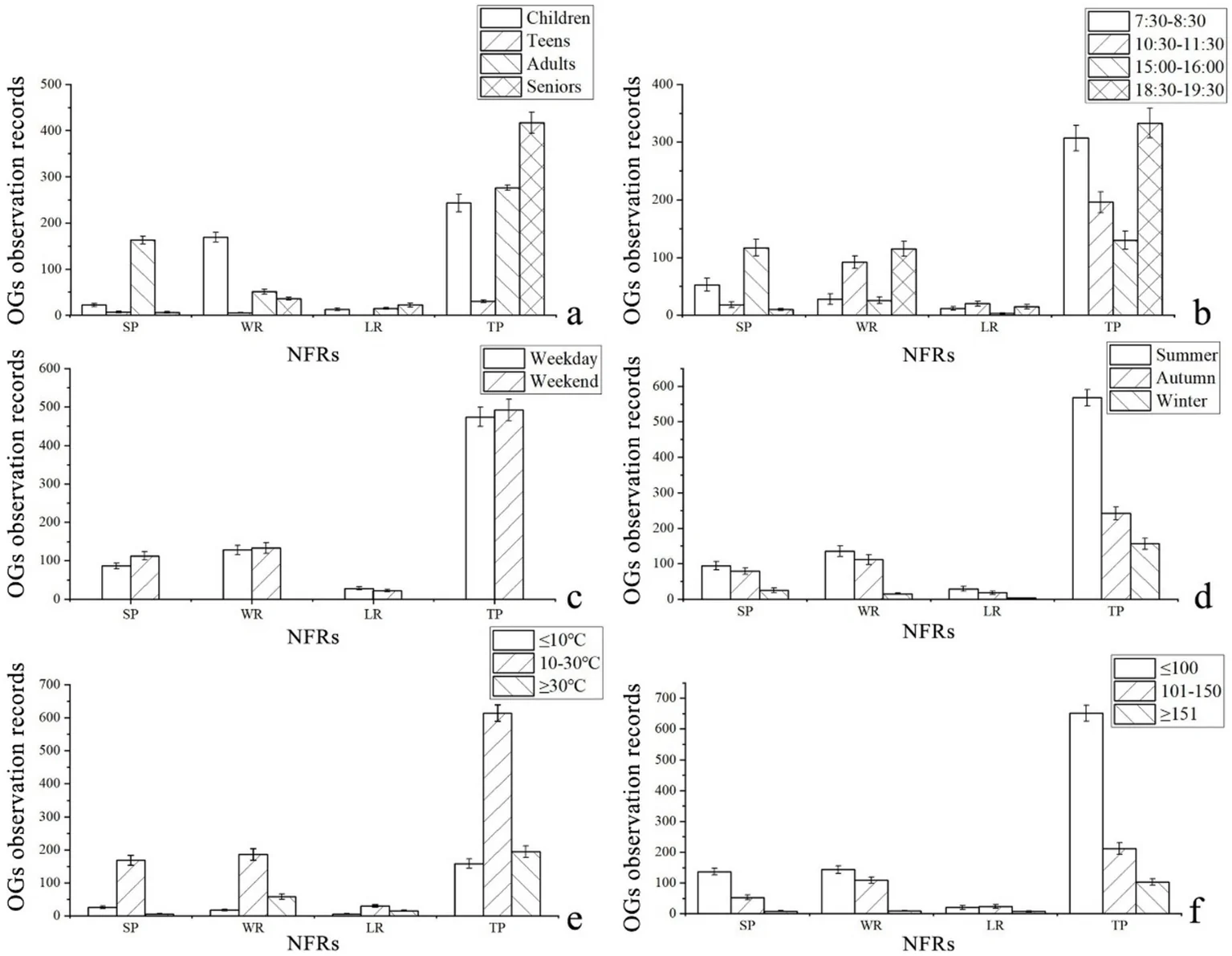
Distribution of OGs observation records in the four NFRs: **(a)** age; **(b)** time period; **(c)** week period; **(d)** season; **(e)** temperature; **(f)** AQI.

The age distribution of OGs observation records varied across the four NFRs (Figure 5a). Adults constituted the largest proportion of OGs observation records at SP, whereas observation records involving seniors were identified at all four NFRs and were most frequent at TP. At WR, children accounted for the largest proportion of OGs observation records, whereas no observation records involving teens were recorded at LR.

The time period distribution of OGs observation records also differed among the four NFRs (Figure 5b). At TP, these observation records were most frequently collected during the morning (07:30–08:30) and evening (18:30–19:30) observation periods, whereas the afternoon period (15:00–16:00) accounted for the highest proportion of OGs observation records at SP. At WR, OGs observation records were most frequently recorded during 18:30–19:30, while at LR the late-morning period (10:30–11:30) accounted for the largest proportion of OGs observation records.

Weekday and seasonal distributions of OGs observation records also varied among the four NFRs. At LR, a greater proportion of OGs observation records was recorded on weekdays, whereas weekend observations predominated at the other three NFRs (Figure 5c). Across all four NFRs, similar seasonal patterns were observed, with the highest number of OGs observation records recorded during summer and the lowest during winter (Figure 5d).

Most OGs observation records were collected when the temperature ranged from 10 to 30 °C across all four NFRs (Figure 5e). Similarly, the majority of OGs observation records were recorded on days with an AQI below 100, except at LR, where a different distribution was observed (Figure 5f).

#### Spatial distribution of observation records across the four NFRs

**Figure 6:**
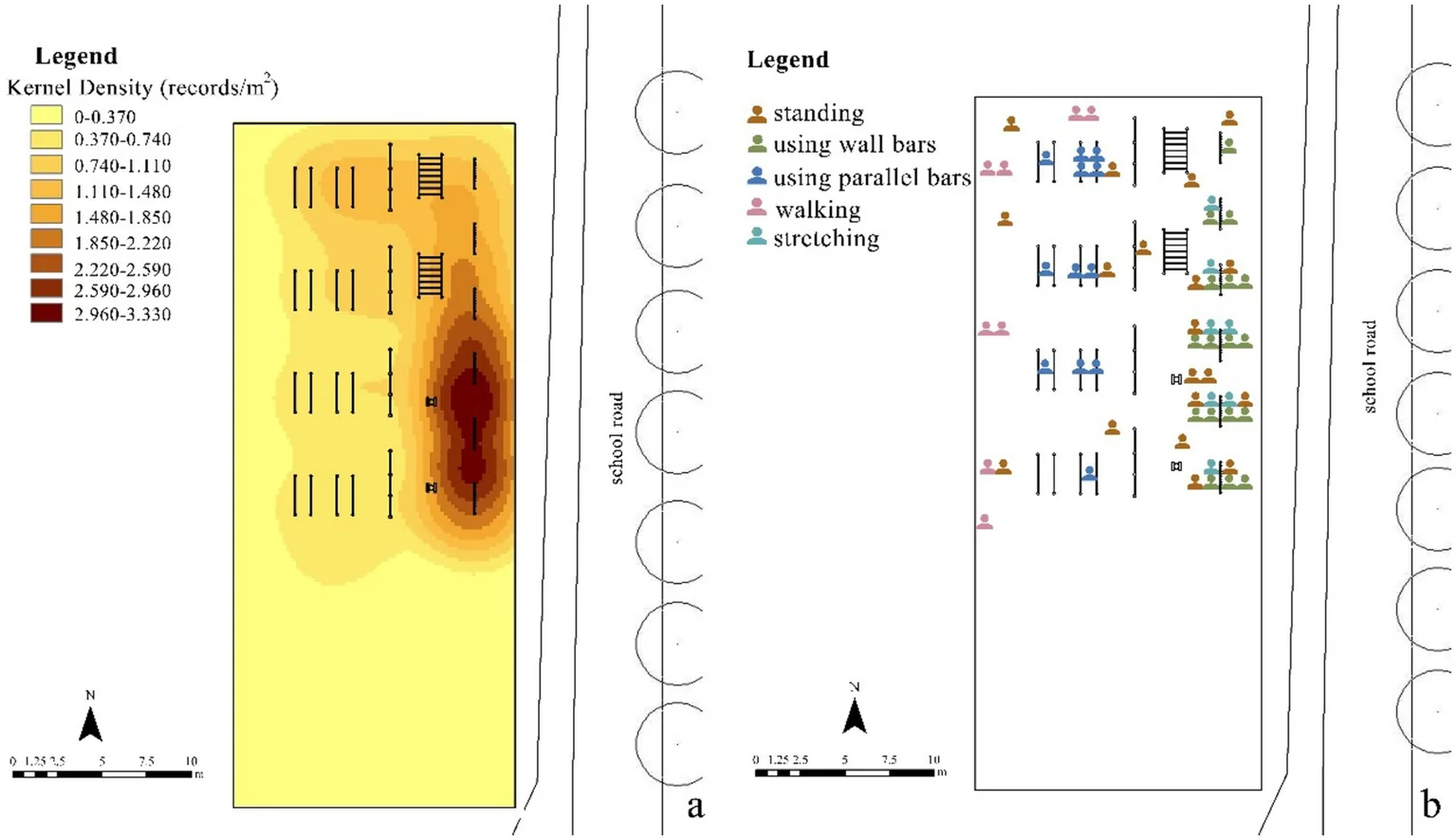
Heat map **(a)** and behavior map **(b)** of observation records at SP. One human icon represents approximately five observation records.

**Figure 7:**
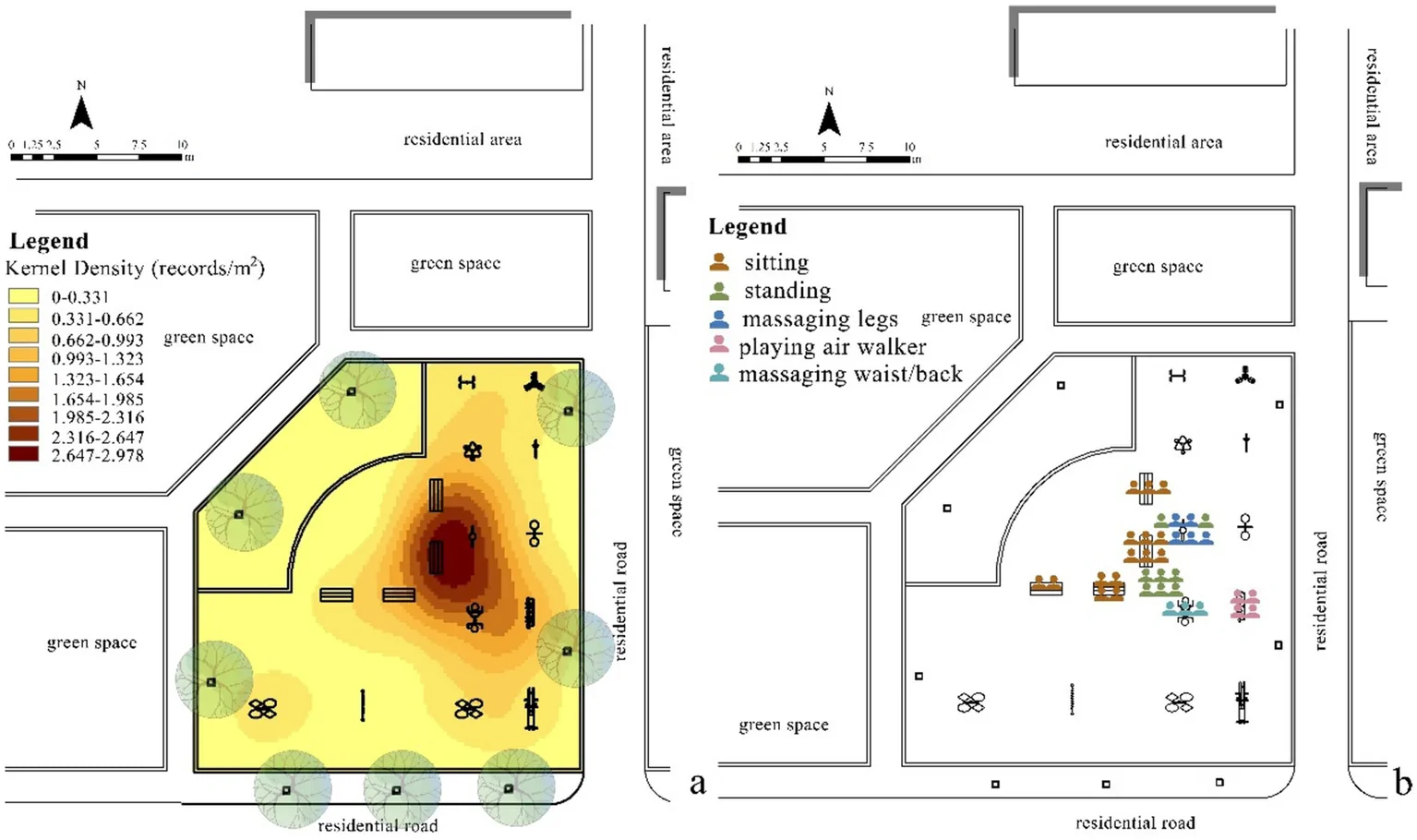
Heat map **(a)** and behavior map **(b)** of observation records at LR. One human icon represents approximately five observation records.

**Figure 8:**
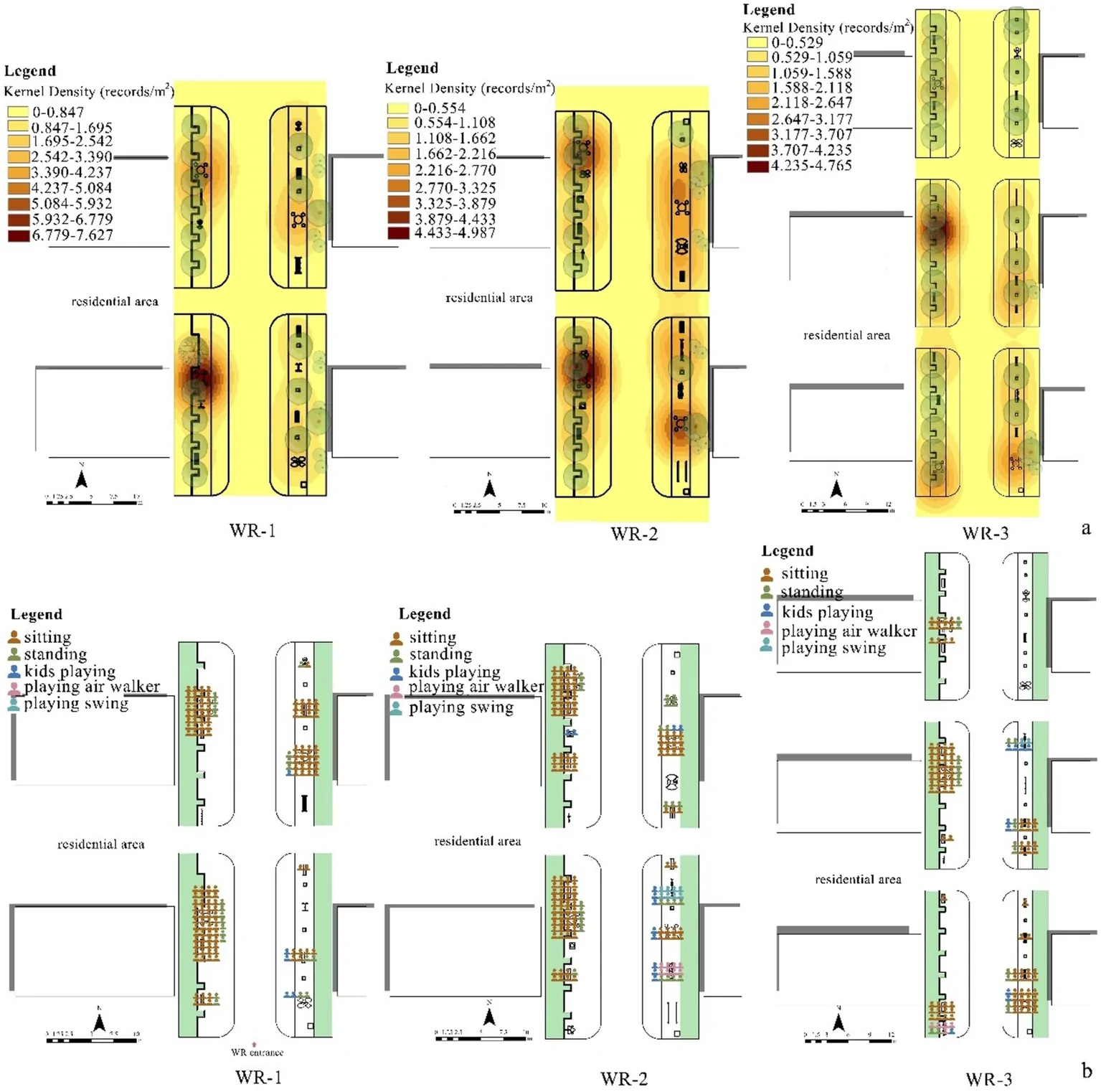
Heat map **(a)** and behavior map **(b)** of observation records at WR. One human icon represents approximately five observation records.

**Figure 9:**
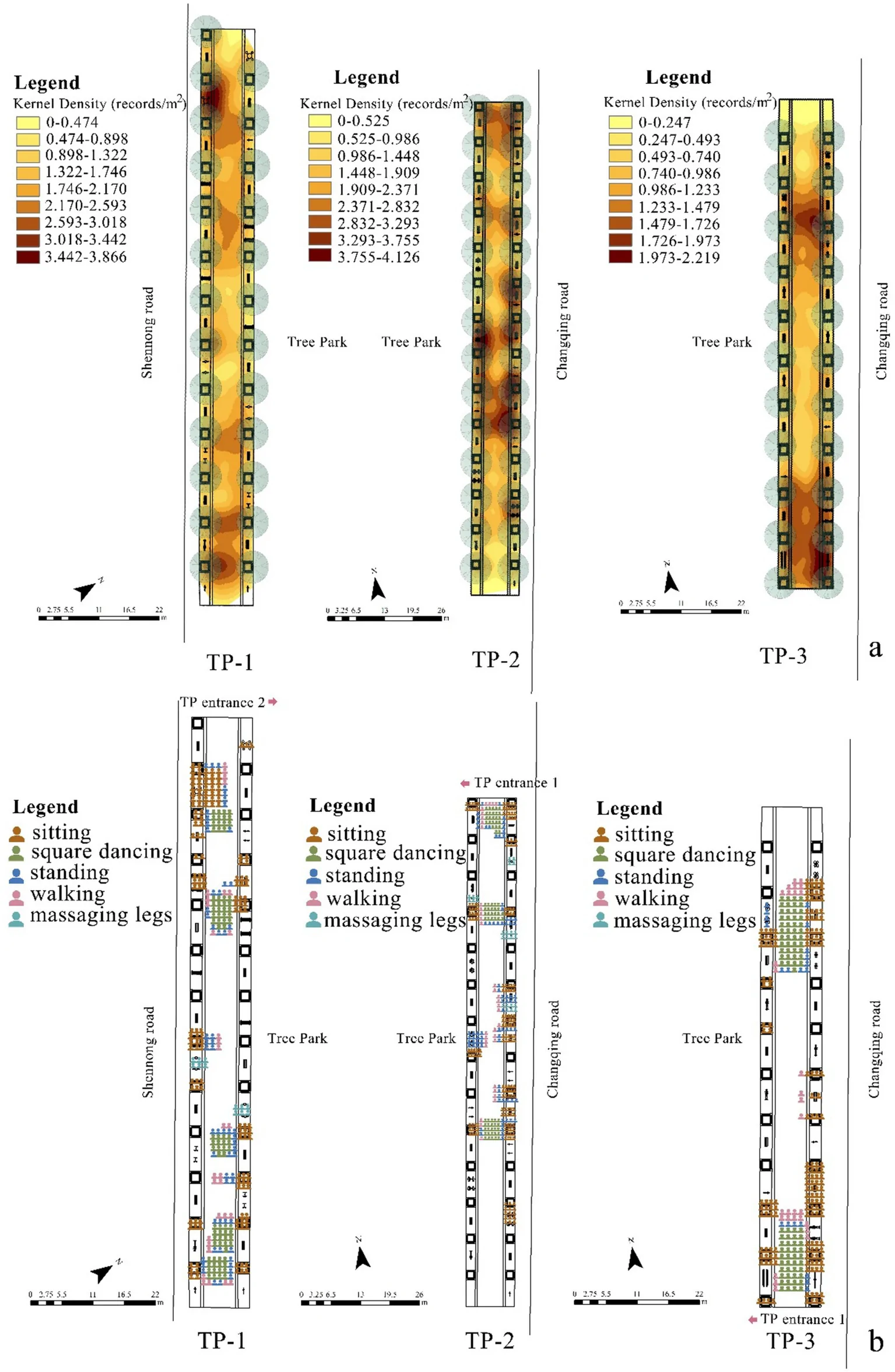
Heat map **(a)** and behavior map **(b)** of observation records at TP. One human icon represents approximately five observation records.

Heat maps and behavior maps were generated to analyze the spatial distribution of observation records across the four NFRs (Figures 6–9). Due to a wide range of behaviors was observed, only the five most frequently recorded behavior categories at each NFR are presented in the behavior maps. In addition, the behavior category “using OGs” was further subdivided according to the type of equipment used.

At SP, the highest activity density (2.960–3.330 records/m^2^; Figure 6a) was concentrated around the wall bars located in the south-eastern section of the site. Unlike the other three NFRs, SP did not contain seating facilities. Standing was the most frequently observed behavior, followed by the using of wall bars, parallel bars, walking, and stretching (Figure 6b).

Standing behavior was distributed throughout the site but was more concentrated in areas with higher observation densities. Observation records involving the use of wall and parallel bars were primarily clustered along the southern edge of the site. Walking was mainly observed along the outer perimeter of the NFR, whereas stretching activities were concentrated around the wall bars. Overall, activity density gradually decreased from east to west and from south to north, with the greatest concentration of observation records occurring in the southeastern area adjacent to the fence and lighting facilities.

At LR, the highest activity density (2.647–2.978 records/m^2^) was concentrated around the seating area (Figure 7a). Sitting was the most frequently observed behavior, followed by standing, using of the leg massager, the air walker and waist/back massager (Figure 7b).

Sitting activities were primarily concentrated near the OGs, particularly around the leg massager and waist/back massager. Standing behavior was mainly observed in areas with higher activity density and around the seating and OGs. Overall, observation records were concentrated in the central activity area where seating and OGs were co-located.

At WR, the highest activity density (6.779–7.627 records/m^2^) was observed at sub-area WR-1 (Figure 8a). By comparison, the maximum density values in WR-2 and WR-3 were lower, reaching 4.987 and 4.765 records/m^2^, respectively. Observation records in WR-1 were primarily concentrated around the seating areas with tables. Sitting was the most frequently observed behavior, followed by standing, kids playing, using the air walker and swinging (Figure 8b).

WR-1, located near the residential entrance, was characterized by a high concentration of sitting activities. Standing behavior was mainly distributed around the perimeter of the seating area, with occasional use of the tai-chi wheel. No air walkers or swings were installed in WR-1.

Kids playing was mainly observed in WR-2 and WR-3, where sculptures and swings were located. Swinging use frequently co-occurred with kids playing, and observation records involving children were commonly concentrated around the air walkers.

Although WR-3 occupied the largest area within WR, it exhibited the lowest activity density. Overall, observation records were concentrated in areas containing seating facilities, tables, and OGs. In addition, shaded areas beneath trees and the semi-enclosed spaces formed by the curved concrete tree planters were associated with higher concentrations of sitting and standing activities, whereas kids playing was primarily observed in the more open spaces on the eastern side of the site.

At TP, the highest activity density (3.755–4.216 records/m^2^) was observed at sub-area TP-2 (Figure 9a). The corresponding maximum density ranges were 3.442–3.866 records/m^2^ in TP-1 and 1.973–2.219 records/m^2^ in TP-3. Sitting was the most frequently observed behavior, followed by square dancing, standing, walking and using of leg massager (Figure 9b).

Unlike the other three NFRs, square dancing was observed exclusively at TP. The central pathway, which was closed to motor vehicles and bicycles, served as the primary location for square dancing. Observation records associated with standing were mainly distributed around the perimeter of the dancing area, with occasional use of the Tai Chi wheel. Walking activities were primarily observed along the outer edge of the gathering area.

Seating facilities and OGs were alternately arranged beneath the trees on both sides of the site, and higher concentrations of observation records were found in these shaded areas. Because no leg massager was installed in TP-3, use of the leg massager was observed only in TP-1 and TP-2. Overall, observation records were more highly concentrated on the side of the site adjacent to the park.

## Discussion

### Age structure of observation records involving OGs use

Adults and seniors accounted for the majority of the observation records across the four NFRs, indicating that these facilities primarily served middle-aged and older populations. This finding is consistent with previous observational studies conducted in Hong Kong and Leipzig, where outdoor fitness stations were primarily used by the seniors (44). In contrast, relatively few observation records involved teens. This pattern may be associated with academic commitments and a preference for indoor fitness facilities that provide opportunities for higher-intensity exercise (32, 45).

The age composition of observation records involving OGs use differed among the four NFRs. Adults accounted for the largest proportion of these observation records at SP, which may reflect the site’s location on a university campus and its primary user population. In contrast, observation records involving seniors were most frequently recorded at TP, consistent with the findings from Asian cities reporting that seniors frequently visit parks for exercise and recreation (7, 9, 10, 44, 46, 47). This finding also agrees with a national survey conducted by the General Administration of Sport of China, which showed that participation in OGs increases with age (48).

Our findings differ from reports from several Western countries, where adults and children account for the largest proportion of OGs use (17, 21, 49, 50). These differences may reflect variations in demographic structure, recreational culture, and the provision of OGs across countries (51). For example, only 4% of users observed in 174 neighborhood parks in the United States were seniors (10). Among the two residential NFRs, observation records involving children accounted for the largest proportion of OGs use at WR, whereas observation records involving seniors predominated at LR. This difference is likely associated with the provision of children’s OGs at WR but not at LR. These findings suggest that age-specific provision of OGs should be considered in future NFR planning to better accommodate the needs of different user groups.

### Behavior characteristics and OGs use

Sitting was the most frequently observed behavior across the four NFRs, accounting for nearly half of all observation records. This result is consistent with previous studies reporting that sedentary activities remain the dominant behavior in urban parks even after the installation of OGs (11). Together with the relatively low proportion of OGs observation records, these findings suggest that NFRs function not only as places for PA, but also as important open spaces for rest and social interaction.

Observation records involving OGs use accounted for 15.2% of all observation records, indicating that although the facilities were regularly used, they did not represent the primary activity within the NFRs. Nevertheless, the proportion was higher than that reported in studies conducted in Los Angeles (17), western Canada (1) and Melbourne (50). One possible explanation is the wider availability of OGs in Chinese residential communities, where seniors may rely more heavily on these freely accessible facilities because of limited alternative opportunities for outdoor exercise (7). In contrast, PA in many Western cities more commonly occurs on lawns, trails, or sports fields (44).

Among the various types of OGs, the air walker, leg massager and waist/back massager were used most frequently. Previous studies have reported inconsistent patterns of equipment preference (52–54), suggesting that utilization is influenced by local demographic characteristics and cultural contexts. The high frequency of rehabilitation-oriented OGs observed in the present study may be related to the large proportion of senior users. Earlier studies have shown that many middle-aged and older adults experience chronic musculoskeletal conditions, particularly lower back pain and knee discomfort, and frequently use OGs for rehabilitation and light exercise (9, 55, 56). These findings highlight the importance of considering the health needs and demographic characteristics of local residents when selecting OGs types.

Behavioral intensity also varied among age groups. Observation records involving children were more frequently classified as MVPA, whereas those involving seniors were predominantly classified as LPA/SB behavior. This finding is consistent with previous research demonstrating age-related differences in PA intensity (57). For many seniors, NFRs may serve not only as places for exercise but also as community spaces for social interaction and recreation (24).

### Spatial distributions of behavioral activities

**Figure 10:**
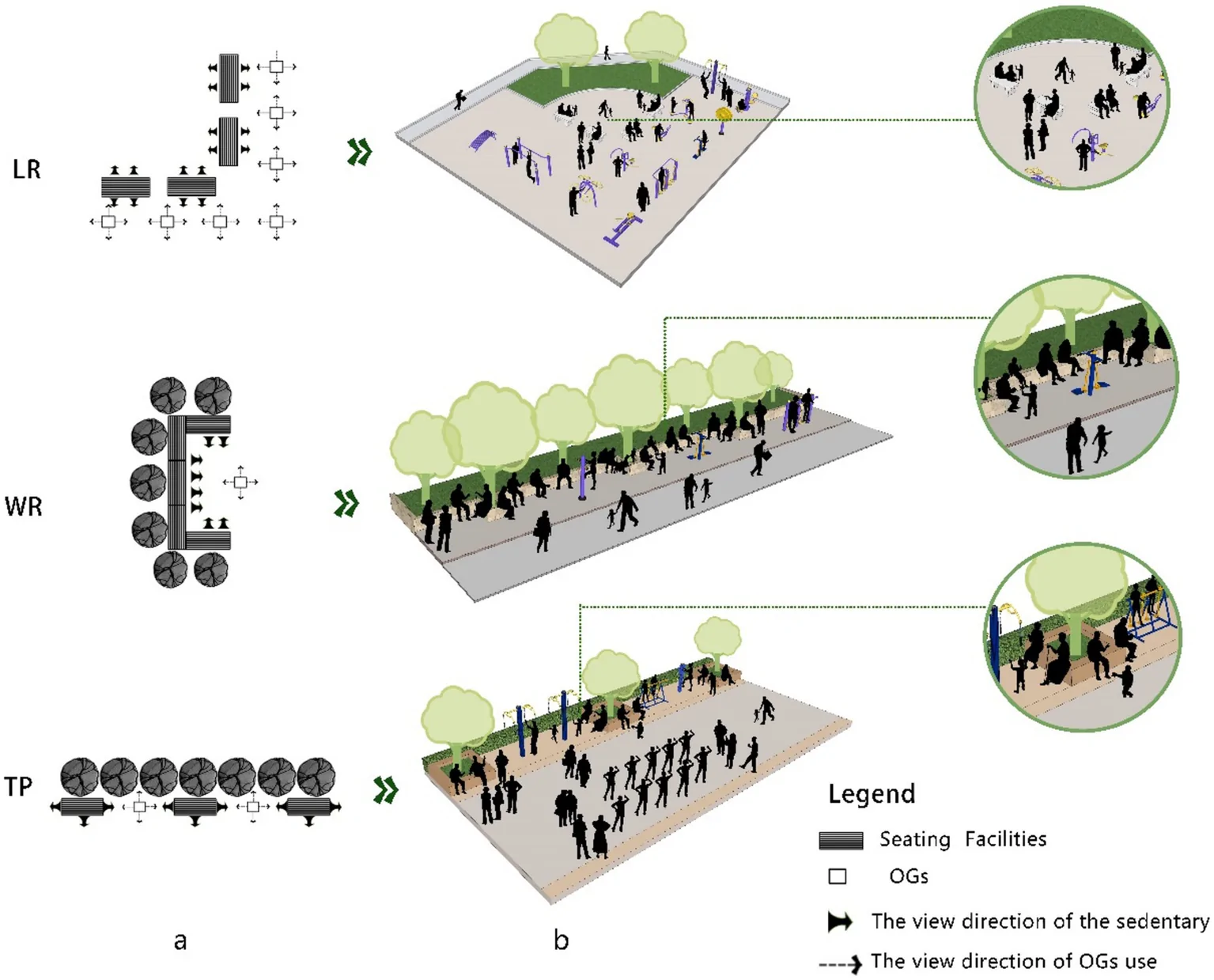
The layout patterns of seats and OGs in LR, WR, and TP **(a)** plan; **(b)** 3D graphs.

Behavioral activities exhibited clear spatial clustering within the four NFRs, with different behaviors consistently associated with specific landscape settings. Sitting was the dominant behavior in LR, WR, and TP and was consistently concentrated in semi-enclosed spaces equipped with seating facilities (Figure 10). Similar findings have been reported in environmental behavior studies, which suggest that semi-enclosed space in UGS provided a greater sense of security and comfort while maintaining visual connections with surrounding activities (58). In WR, for example, sitting activities were concentrated in the shaded semi-enclosed spaces formed by trees and curved planting beds, whereas the adjacent open spaces were primarily used for kids playing.

Standing behavior was more broadly distributed but were mainly concentrated around areas with high activity density, such as square dancing, OGs and seating areas. Previous studies have suggested that active public spaces often attract additional observers and informal social interaction (59). The concentration of standing behavior around dancing groups and OGs in the present study further supports the role of these locations as social gathering spaces rather than merely exercise facilities.

Walking was the predominant PA in TP and SP. The continuous pathways, separation from motor vehicles, and shaded walking environments may have supported walking activities, particularly among seniors. This interpretation is consistent with recent evidence from Chengdu, China, showing that sidewalk provision, population density, and access to medical facilities were positively associated with older adults’ walking time, while these associations varied across spatial locations and levels of walking time (8). Together with previous studies reporting associations between accessible walking environments and recreational walking (7, 60–63), these findings highlight the importance of considering both the availability and spatial configuration of walking-supportive environments when planning NFRs. Square dancing, observed exclusively in TP, was concentrated within the open central pathway, where the combination of unobstructed space and adjacent seating appeared suitable for organized group activities.

Kids playing was primarily concentrated in WR, where sculptures and swings were installed. Previous studies have shown that play facilities and interactive landscape elements can increase children’s engagement with public open spaces (9, 32, 64). During observations, children were also frequently seen using OGs in unintended ways, particularly treadmill-type equipment. Although this behavior may reflect the attractiveness of the facilities, it may also increase the risk of injury. Therefore, age-appropriate equipment selection, clear safety guidance, and appropriate supervision should be considered in future NFR design and management to improve both safety and usability.

### Factors associated with MVPA

The multivariable logistic regression analysis showed that age, time period, week period, temperature, AQI, and site characteristics were independently associated with MVPA, whereas gender and season were not significantly associated with MVPA.

Observation records collected during the morning (07:30–08:30) were associated with higher odds of MVPA than those collected during the evening reference period (18:30–19:30), whereas records collected at 10:30–11:30 and particularly at 15:00–16:00 were less likely to be classified as MVPA. This finding agrees with previous studies comparing park use in China and Germany, which reported higher levels of morning PA among Chinese park users (44, 65), possibly reflecting more favorable environmental conditions and daily exercise routines.

Season was not independently associated with MVPA, suggesting that the observed seasonal differences were largely explained by other demographic, environmental, and site-related factors. In contrast, weekday observation records showed lower odds of MVPA than weekend records, indicating that higher-intensity PA was more likely to occur during weekends.

Environmental conditions were also associated with MVPA. Observation records collected when AQI was ≤100 showed higher odds of MVPA than those collected under poor air quality, suggesting that favorable air quality may encourage higher-intensity PA.

Site characteristics were also associated with activity intensity. Observation records from SP exhibited higher odds of MVPA, whereas those from WR exhibited lower odds. These differences may be associated with variations in facility configuration and spatial design. SP primarily contained power machines and lacked conventional seating facilities, whereas WR included extensive seating areas and a roadway crossing the site, both of which may have encouraged lower-intensity activities. These findings suggest that the spatial configuration and functional composition of NFRs may contribute to differences in the intensity of observed PA.

### Implications for NFRs planning and design

The present findings provide several practical implications for the planning and management of NFRs. First, NFRs located within residential communities may promote greater use of OGs because of their accessibility for nearby residents. Although TP accommodated the largest number of observation records, the highest proportion of OGs use was observed in LR, highlighting the importance of proximity to residential populations for facility utilization.

Second, the spatial arrangement of OGs, seating facilities, pathways, and vegetation appears to influence activity patterns. Semi-enclosed seating areas adjacent to OGs supported both resting and social interaction, whereas open spaces facilitated group activities such as square dancing and kids playing. Integrating multiple complementary activity spaces within the same NFR may therefore support a wider range of recreational behaviors.

Third, OGs should be selected according to the demographic characteristics and potential health needs of local residents. Rehabilitation-oriented equipment may better serve communities with larger seniors populations, whereas children’s fitness and play facilities may be more appropriate in family-oriented neighborhoods. Because some equipment was occasionally used incorrectly by children, appropriate safety signage and educational guidance should also be incorporated into NFRs management strategies.

Overall, these findings support the importance of combining age-appropriate facilities, accessible walking environments, diverse activity spaces, and comfortable resting areas to promote both PA and social interaction within NFRs.

## Strengths and limitations

This study has several strengths. First, it combined systematic SOPARC observations with kernel density estimation and behavior mapping to examine the spatial distribution of activities within NFRs. The integration of observational and spatial analytical methods provides a more comprehensive understanding of the relationships between landscape design and human behavior than either method alone. Second, a binary logistic regression was used to identify factors associated with MVPA, providing quantitative evidence to support future planning and design decisions.

Several limitations should be acknowledged. First, SOPARC records person-observations rather than individually identifiable participants; therefore, some individuals may have been observed repeatedly across different sessions, and the findings should be interpreted as patterns of observed use rather than estimates of unique users. Second, SOPARC captures instantaneous behaviors and cannot measure activity duration, activity frequency, user motivations, or other socio-demographic characteristics. Future studies could combine behavioral observation with questionnaires or wearable devices to obtain more comprehensive information. Third, this observational study included only four NFRs with different functions, sizes, and user populations in a single city and covered three seasons. Consequently, the findings should be interpreted as associations rather than causal effects, and caution is warranted when generalizing them to other settings. Finally, PA intensity was classified according to observed behavior types rather than direct physiological measurements, and the study sites represented a relatively limited range of landscape characteristics. Future multi-site, longitudinal, or intervention-based studies are needed to validate these findings and further examine how landscape design relates to PA and OGs use.

## Conclusion

The installation of NFRs has become an increasingly important component of UGS in China, supporting the implementation of the National Fitness Strategy and expanding access to public fitness facilities. By integrating systematic *in situ* observation, behavior mapping, kernel density estimation, and logistic regression analysis, this study provides a comprehensive understanding of how NFRs are used and how their spatial characteristics relate to observed PA.

Our findings demonstrate that NFRs should not be regarded solely as exercise facilities, but as multifunctional public spaces that simultaneously support PA, recreation, and social interaction. Rather than the quantity of OGs alone, the overall spatial configuration of NFRs—including the organization of OGs, walking environments, seating, vegetation, and flexible activity spaces—appears to contribute to differences in observed activity patterns and active use.

These findings highlight the need to shift future NFR planning from an equipment-oriented approach toward a human-centered and behavior-oriented design strategy. Integrating age-appropriate OGs with supportive landscape elements and diverse activity spaces may better accommodate the needs of different population groups while creating healthier, safer, and more inclusive public environments.

Overall, this study provides evidence-based insights into the relationships between landscape design, OGs, and human behavior in NFRs. These findings may inform landscape architects, urban planners, and public health practitioners in designing healthier, safer, and more inclusive public fitness environments under China’s National Fitness Strategy. Given the observational design and the limited number of study sites, the findings should be interpreted as evidence of associations rather than causal effects and warrant further validation in a broader range of NFR settings.

## Data Availability

The raw data supporting the conclusions of this article will be made available by the authors, without undue reservation.

## References

[1] Copeland JL Currie C Walker A Mason E Willoughby TN Amson A. Fitness equipment in public parks: frequency of use and community perceptions in a small urban centre. J Phys Act Health. (2017) 14:344–52. doi: 10.1123/jpah.2016-0277, 28169574

[2] WHO. Physical inactivity: a global public health problem. Geneva: World Health Organization (2020).

[3] Akpinar A. How is quality of urban green spaces associated with physical activity and health. Urban For Urban Green. (2016) 16:76–83. doi: 10.1016/j.ufug.2016.01.011

[4] Kaczynski AT Potwarka LR Saelens BE. Association of park size, distance, and features with physical activity in neighborhood parks. Am J Public Health. (2008) 98:1451–6. doi: 10.2105/AJPH.2007.129064, 18556600 PMC2446450

[5] Wang H Dai X Wu J Wu X Nie X. Influence of urban green open space on residents' physical activity in China. BMC Public Health. (2019) 19:1093. doi: 10.1186/s12889-019-7416-7, 31409316 PMC6693084

[6] Akpinar A Cankurt M. How are characteristics of urban green space related to levels of physical activity: examining the links. Indoor Built Environ. (2017) 26:1091–101. doi: 10.1177/1420326x16663289

[7] Zhai Y Li D Wu C Wu H. Urban park facility use and intensity of seniors' physical activity -an examination combining accelerometer and GPS tracking. Landsc Urban Plan. (2021) 205:103950. doi: 10.1016/j.landurbplan.2020.103950

[8] Wei D Chen J Jiang YX Yang LC. Examining the associations between the built environment and older adults’ walking behavior: spatial and distributional heterogeneity. Int J Sustain Transp. (2026) 20:305–18. doi: 10.1080/15568318.2025.2573742

[9] Cohen DA Han B Williamson S Nagel C McKenzie TL Evenson KR . Playground features and physical activity in US neighborhood parks. Prev Med. (2020) 131:105945. doi: 10.1016/j.ypmed.2019.105945, 31805315 PMC7405885

[10] Chow HW Mowen AJ Wu GL. Who is using outdoor fitness equipment and how? The case of Xihu Park. Int J Environ Res Public Health. (2017) 14:448., 28430141 10.3390/ijerph14040448 PMC5409648

[11] Cranney L Phongsavan P Kariuki M Stride V Scott A Hua M . Impact of an outdoor gym on park users' physical activity: a natural experiment. Health Place. (2016) 37:26–34. doi: 10.1016/j.healthplace.2015.11.002, 26699448

[12] Sami M Smith M Ogunseitan OA. Placement of outdoor exercise equipment and physical activity: a quasi-experimental study in two parks in Southern California. Int J Environ Res Public Health. (2020) 17:2605. doi: 10.3390/ijerph17072605, 32290320 PMC7178161

[13] Marcos-Pardo PJ Espeso-García A Abelleira-Lamela T Machado DRL. Optimizing outdoor fitness equipment training for older adults: benefits and future directions for healthy aging. Exp Gerontol. (2023) 181:112279. doi: 10.1016/j.exger.2023.112279, 37611645

[14] Sami M Smith M Ogunseitan OA. Changes in physical activity after installation of a fitness zone in a community park. Prev Chronic Dis. (2018) 15:9. doi: 10.5888/pcd15.170560, 30095404 PMC6093269

[15] Levinger P Panisset M Parker H Batchelor F Tye M Hill KD. Guidance about age-friendly outdoor exercise equipment and associated strategies to maximise usability for older people. Health Promot J Austr. (2021) 32:475–82. doi: 10.1002/hpja.367, 32484939 PMC8359243

[16] Sharma R Chaudhary M. Perceptions of outdoor gymnasiums in National Capital Region, India: creating active environments for health promotion. Health Promot Int. (2021) 36:89–100. doi: 10.1093/heapro/daaa028, 32337575

[17] Cohen DA Marsh T Williamson S Golinelli D McKenzie TL. Impact and cost-effectiveness of family fitness zones: a natural experiment in urban public parks. Health Place. (2012) 18:39–45. doi: 10.1016/j.healthplace.2011.09.008, 22243905 PMC3308725

[18] Kim DI Lee DH Hong S Jo SW Won YS Jeon JY. Six weeks of combined aerobic and resistance exercise using outdoor exercise machines improves fitness, insulin resistance, and chemerin in the Korean elderly: a pilot randomized controlled trial. Arch Gerontol Geriatr. (2018) 75:59–64. doi: 10.1016/j.archger.2017.11.006, 29190545

[19] Sales M Polman R Hill KD Levinger P. A novel exercise initiative for seniors to improve balance and physical function. J Aging Health. (2017) 29:1424–43. doi: 10.1177/0898264316662359, 27511957

[20] Grigoletto A Mauro M Maietta Latessa P Iannuzzi V Gori D Campa F . Impact of different types of physical activity in green urban space on adult health and behaviors: a systematic review. Eur J Invest Health Psychol Educ. (2021) 11:263–75. doi: 10.3390/ejihpe11010020, 34542463 PMC8314339

[21] Bettencourt L Neves R. Seniors' playground and physical activity: perceptions and practices. J Aging Phys Act. (2021) 20:S276–7. doi: 10.13140/2.1.3203.1684

[22] Aparicio EH Rodríguez E Marbán R Minguet J. Analysis of the public geriatric parks for elderly people in Málaga (Spain). Retos Nuevas Perspectivas de Educación Física Deporte y Recreación. (2010) 17:99–102.

[23] Mora R. Moving bodies: open gyms and physical activity in Santiago. J Urban Des. (2012) 17:485–97. doi: 10.1080/13574809.2012.706367

[24] Chow HW. Outdoor fitness equipment in parks: a qualitative study from older adults' perceptions. BMC Public Health. (2013) 13:1216. doi: 10.1186/1471-2458-13-1216, 24359536 PMC3878060

[25] Ferreira KRA Ahmadi S Sampaio RAC Uchida MC. Outdoor gyms and physical function: a cross-sectional comparative study between active and sedentary older adults. J Bodyw Mov Ther. (2023) 33:76–81. doi: 10.1016/j.jbmt.2022.09.018, 36775529

[26] Dai X Dong Q. Study on spatial factors of national fitness route-report of an environment and behavior study on five sites in Hangzhou. Chin Landsc Archit. (2015) 31:101–5.

[27] General Administration of Sport of China (2025). Data from the 2024 National Survey of Sports Venue Statistics. Beijing: General Administration of Sport of China. Available online at: https://www.gov.cn/lianbo/bumen/202503/content_7014540.htm (Accessed March 19, 2025)

[28] General Administration of Sport of China (2023). Work Programme of the Action to Enhance National Fitness Facilities (2023–2025). Beijing: General Administration of Sport of China. Available online at: https://www.gov.cn/zhengce/zhengceku/202306/content_6884623.htm (Accessed October 20, 2024)

[29] Bertiato F. The ‘motor paradox’ of outdoor fitness spaces. Cities. (2025) 158:105650. doi: 10.1016/j.cities.2024.105650

[30] Hsueh-Wen C Chia-Hua H Francesca PM. Does the use of outdoor fitness equipment by older adults qualify as moderate to vigorous physical activity? PLoS One. (2018) 13:e0196507. doi: 10.1371/journal.pone.0196507 29709035 PMC5927431

[31] Cunha FA Gomes GSM Carvalho J da Silva NSL. Concurrent exercise circuit protocol performed in public fitness facilities meets the American College of Sports Medicine guidelines for energy cost and metabolic intensity among older adults in Rio de Janeiro City. Appl Physiol Nutr Metab. (2019) 44:477–84. doi: 10.1139/apnm-2018-0513, 30273500

[32] Mu B Liu C Mu T Xu X Tian G Zhang Y . Spatiotemporal fluctuations in urban park spatial vitality determined by on-site observation and behavior mapping: a case study of three parks in Zhengzhou City, China. Urban For Urban Green. (2021) 64:127246. doi: 10.1016/j.ufug.2021.127246

[33] Liu K Siu WM Gong XY Gao Y Lu D. Where do networks really work? The effects of the Shenzhen greenway network on supporting physical activities. Landsc Urban Plan. (2016) 152:49–58. doi: 10.1016/j.landurbplan.2016.04.001

[34] Çamaş NÇ Erçek YD Dumitrescu RI Atay Kaya İ. Urban design requirements promoting the behaviours of children and parents in school squares with different spatial characteristics. Cities. (2025) 165:106084. doi: 10.1016/j.cities.2025.106084

[35] Cosco NG Moore RC Islam MZ. Behavior mapping: a method for linking preschool physical activity and outdoor design. Med Sci Sports Exerc. (2010) 42:513–9. doi: 10.1249/MSS.0b013e3181cea27a, 20068497

[36] Park K Christensen K Lee D. Unmanned aerial vehicles (UAVs) in behavior mapping: a case study of neighborhood parks. Urban For Urban Green. (2020) 52:126693. doi: 10.1016/j.ufug.2020.126693

[37] Cox A Loebach J Little S. Understanding the nature play milieu: using behavior mapping to investigate children's activities in outdoor play spaces. Child Youth Environ. (2018) 28:232–61. doi: 10.1353/cye.2018.0018

[38] Refshauge AD Stigsdotter UD Petersen LS. Play and behavior characteristics in relation to the design of four Danish public playgrounds. Child Youth Environ. (2013) 23:22–48. doi: 10.1353/cye.2013.0040

[39] Phillips EK Dave MG Ashe MC Schultz ASH O’Keefe-McCarthy S Arora RC . Mobility in a cardiac surgery intensive care unit: a behaviour mapping study. Intensive Crit Care Nurs. (2025) 87:103918. doi: 10.1016/j.iccn.2024.103918, 39733665

[40] Ministry of Housing and Urban-rural Development of China (2017). Standard for Classification of Urban Green Space. Beijing: Ministry of Housing and Urban-Rural Development of the People’s Republic of China. Available online at: http://www.mohurd.gov.cn/wjfb/201806/t20180626_236545.html (Accessed October 29, 2021)

[41] Bureau of Statistics of Shaanxi Province. Shaanxi Statistical Yearbook 2024. Beijing: China Statistics Press (2024).

[42] Wang X Tang P He Y Woolley H Hu X Yang L . The correlation between children's outdoor activities and community space characteristics: a case study utilizing SOPARC and KDE methods in Chengdu, China. Cities. (2024) 150:105002. doi: 10.1016/j.cities.2024.105002

[43] Ainsworth BE Haskell WL Herrmann SD Meckes N Bassett DR Jr Tudor-Locke C . 2011 Compendium of physical activities: a second update of codes and MET values. Med Sci Sports Exerc, 2011. (2011) 43:1575–81. doi: 10.1249/MSS.0b013e31821ece12 21681120

[44] Duan Y Petra W Ru Z Hagen W Walter B. Physical activity areas in urban parks and their use by the elderly from two cities in China and Germany. Landsc Urban Plan. (2018) 178:261–9. doi: 10.1016/j.landurbplan.2018.06.009

[45] Kim Y Shin GS Park J Kang M Son K Kim YM . Effects of multidisciplinary health promotion program among children in community childcare center. Clin Nutr Res. (2024) 13:8–21. doi: 10.7762/cnr.2024.13.1.8, 38362127 PMC10866676

[46] Shan XZ. Socio-demographic variation in motives for visiting urban green spaces in a large Chinese city. Habitat Int. (2014) 41:114–20. doi: 10.1016/j.habitatint.2013.07.012

[47] Zhai Y Li D Wang D Shi C. Seniors' physical activity in neighborhood parks and park design characteristics. Front Public Health. (2020) 8:322. doi: 10.3389/fpubh.2020.00322, 32850576 PMC7403175

[48] General Administration of Sport of China (2015). State of the Nation's Fitness Activities Survey Bulletin 2014. Beijing: General Administration of Sport of China. Available online at: http://www.sport.gov.cn/n316/n340/c212777/content.html (Accessed October 20, 2021)

[49] Jansson A Lubans D uncan MD Plotnikoff M Smith J Robards S . An observational study of outdoor gym features, usage and user characteristics. J Sci Med Sport. (2019) 22:S102–3. doi: 10.1016/j.jsams.2019.08.134 31444034

[50] Veitch J Salmon J Abbott G Timperio A Sahlqvist S. Understanding the impact of the installation of outdoor fitness equipment and a multi-sports court on park visitation and park-based physical activity: a natural experiment. Health Place. (2021) 71:102662. doi: 10.1016/j.healthplace.2021.102662, 34517270

[51] Ma CY Yang C. The moderating effects of mobile applications on the use of urban green space and mental health of older people: a mixed-method investigation in Hong Kong. Urban For Urban Green. (2024) 91:128182. doi: 10.1016/j.ufug.2023.128182

[52] Jansson AK Lubans DR Smith JJ Duncan MJ Haslam R Plotnikoff RC. A systematic review of outdoor gym use: current evidence and future directions. J Sci Med Sport. (2019) 22:1335–43. doi: 10.1016/j.jsams.2019.08.003, 31444034

[53] Jansson AK Lubans DR Duncan MJ Smith JJ Bauman A Attia J . Increasing participation in resistance training using outdoor gyms: a study protocol for the ecofit type III hybrid effectiveness implementation trial. Contemp Clin Trials Commun. (2024) 41:101358. doi: 10.1016/j.conctc.2024.101358, 39280786 PMC11399599

[54] Janet L Temmy L Rainbow H. Understanding outdoor gyms in public open spaces: a systematic review and integrative synthesis of qualitative and quantitative evidence. Int J Environ Res Public Health. (2018) 15:590. doi: 10.3390/ijerph15040590 29587402 PMC5923632

[55] Huang Z Shi X Li X Zhang L Wu P Mao J . Network pharmacology approach to uncover the mechanism governing the effect of Simiao powder on knee osteoarthritis. Biomed Res Int. (2020) 2020:6971503–3. doi: 10.1155/2020/6971503, 33376732 PMC7738782

[56] Zhu H Chen G Wang Y Lin X Zhou J Wang Z . Dimethyl fumarate protects nucleus pulposus cells from inflammation and oxidative stress and delays the intervertebral disc degeneration. Exp Ther Med. (2020) 20:269–9. doi: 10.3892/etm.2020.9399, 33199994 PMC7664592

[57] Motomura M Koohsari MJ Ishii K Shibata A Nakaya T Hanibuchi T . Park proximity and older adults’ physical activity and sedentary behaviors in dense urban areas. Urban For Urban Green. (2024) 95:128275. doi: 10.1016/j.ufug.2024.128275, 42574925

[58] Naghibi M Faizi M Ekhlassi A. Design possibilities of leftover spaces as a pocket park in relation to planting enclosure. Urban For Urban Green. (2021) 64:127273. doi: 10.1016/j.ufug.2021.127273

[59] Shi SL. Important elements and features of neighborhood landscape for aging in place: a study in Hong Kong. Front Public Health. (2020) 8:316–6. doi: 10.3389/fpubh.2020.00316, 33014951 PMC7461922

[60] McCormack GR Patterson M Frehlich L Lorenzetti DL. The association between the built environment and intervention-facilitated physical activity: a narrative systematic review. Int J Behav Nutr Phys Act. (2022) 19:86. doi: 10.1186/s12966-022-01326-9, 35836196 PMC9284898

[61] Zhai Y Baran PK. Urban park pathway design characteristics and senior walking behavior. Urban For Urban Green. (2017) 21:60–73. doi: 10.1016/j.ufug.2016.10.012

[62] Lu ZP. Investigating walking environments in and around assisted living facilities: a facility visit study. HERD. (2010) 3:58–74. doi: 10.1177/193758671000300406, 21165852

[63] Stride V Cranney L Scott A Hua M. Outdoor gyms and older adults - acceptability, enablers and barriers: a survey of park users. Health Promot J Austr. (2017) 28:243–6. doi: 10.1071/HE16075, 28264761

[64] Veitch J Ball K Flowers E Deforche B Timperio A. Children's ratings of park features that encourage park visitation, physical activity and social interaction. Urban For Urban Green. (2021) 58:126963. doi: 10.1016/j.ufug.2020.126963

[65] Tu H Liao X Schuller K Cook A Fan S Lan G . Insights from an observational assessment of park-based physical activity in Nanchang, China. Prev Med Rep. (2015) 2:930–4. doi: 10.1016/j.pmedr.2015.08.022, 26844171 PMC4721293

